# Framework for developing a national surgical, obstetric and anaesthesia plan

**DOI:** 10.1002/bjs5.50190

**Published:** 2019-07-24

**Authors:** K. A. Sonderman, I. Citron, S. Mukhopadhyay, K. Albutt, K. Taylor, D. Jumbam, K. R. Iverson, M. Nthele, A. Bekele, E. Rwamasirabo, S. Maongezi, M. L. Steer, R. Riviello, W. Johnson, J. G. Meara

**Affiliations:** ^1^ Program in Global Surgery and Social Change, Harvard Medical School, Boston, Massachusetts, USA; ^2^ Center for Surgery and Public Health, Brigham and Women's Hospital, Boston, Massachusetts, USA; ^3^ Department of Surgery, Massachusetts General Hospital, Boston, Massachusetts, USA; ^4^ Department of Plastic and Oral Surgery, Boston Children's Hospital, Boston, Massachusetts, USA; ^5^ Zambian Ministry of Health, Lusaka, Zambia; ^6^ School of Medicine, Addis Ababa University, Addis Ababa, Ethiopia; ^7^ King Faisal Hospital/Oshen, Rwanda Surgical Society, Kigali, Rwanda; ^8^ Tanzania Ministry of Health, Community Development, Gender, Elderly, and Children, Dodoma, Tanzania; ^9^ Emergency and Essential Surgical Care Programme, World Health Organization, Geneva, Switzerland

## Abstract

**Background:**

Emergency and essential surgical, obstetric and anaesthesia (SOA) care are now recognized components of universal health coverage, necessary for a functional health system. To improve surgical care at a national level, strategic planning addressing the six domains of a surgical system is needed. This paper details a process for development of a national surgical, obstetric and anaesthesia plan (NSOAP) based on the experiences of frontline providers, Ministry of Health officials, WHO leaders, and consultants.

**Methods:**

Development of a NSOAP involves eight key steps: Ministry support and ownership; situation analysis and baseline assessments; stakeholder engagement and priority setting; drafting and validation; monitoring and evaluation; costing; governance; and implementation. Drafting a NSOAP involves defining the current gaps in care, synthesizing and prioritizing solutions, and providing an implementation and monitoring plan with a projected cost for the six domains of a surgical system: infrastructure, service delivery, workforce, information management, finance and governance.

**Results:**

To date, four countries have completed NSOAPs and 23 more have committed to development. Lessons learned from these previous NSOAP processes are described in detail.

**Conclusion:**

There is global movement to address the burden of surgical disease, improving quality and access to SOA care. The development of a strategic plan to address gaps across the SOA system systematically is a critical first step to ensuring countrywide scale‐up of surgical system‐strengthening activities.

## Abstract (es)

**Antecedentes:**

En la actualidad, se reconoce que la atención quirúrgica, obstétrica y anestésica urgente y esencial (*surgical, obstetric, and anaesthesia*, SOA) es uno de los componentes de la cobertura sanitaria universal y un elemento necesario para el funcionamiento de un sistema de salud. Para mejorar la atención quirúrgica a nivel nacional, se necesita una planificación estratégica que aborde los seis dominios de un sistema quirúrgico. En este artículo, se detalla el proceso para el desarrollo de un plan nacional de cirugía, obstetricia y anestesia (*national surgical, obstetric, and anaesthesia plan*, NSOAP) basado en las experiencias de los principales proveedores, los funcionarios del Ministerio de Salud, los líderes de la Organización Mundial de la Salud y consultores.

**Métodos:**

El desarrollo de un NSOAP incluye ocho pasos clave: (1) apoyo y dependencia del ministerio, (2) análisis de la situación y evaluaciones de referencia, (3) compromiso de los agentes implicados y establecimiento de prioridades, (4) redacción y validación, (5) seguimiento y evaluación, (6) análisis de costes, (7) gobernanza y (8) implementación. Redactar un NSOAP implica definir los déficits actuales en la atención, sintetizar y priorizar soluciones, y proporcionar un plan de implementación y seguimiento con unos costes proyectados para los seis dominios de un sistema quirúrgico: infraestructura, prestación de servicios, personal, gestión de la información, finanzas y gobernanza.

**Resultados:**

Hasta la fecha, cuatro países han completado un NSOAP y 23 más se han comprometido con su desarrollo. Las lecciones aprendidas de estos procesos previos de NSOAP se describen con detalle.

**Conclusiones:**

Existe un movimiento global para abordar la carga de las enfermedades que precisan cirugía, mejorar la calidad y el acceso a la atención SOA. El desarrollo de un plan estratégico para la aproximación sistemáticamente los déficits en todo el sistema SOA es un primer paso crítico para garantizar la ampliación a nivel nacional de las actividades de fortalecimiento del sistema quirúrgico.

## Introduction

The United Nations and WHO have made universal health coverage (UHC) a clear priority through the Sustainable Development Goals (SDGs)^1^. UHC is defined as all people and communities having access to the promotive, preventive, curative, rehabilitative and palliative health services they need, of sufficient quality to be effective, while also ensuring that the user of these services is not exposed to financial hardship^2^. The World Bank Group also recognizes UHC as key to achieving its goals of ending extreme poverty and increasing equity and shared prosperity^3^. Emergency and essential surgical and anaesthesia care are core components of UHC and a functional health system, but significant gaps exist. The SDG target is 80 per cent UHC coverage by 2030^4^, ^5^. In 2015, the Lancet Commission on Global Surgery^5^ defined the landscape of surgical care worldwide and laid out an initial framework for how low‐ and middle‐income countries could address the gaps in care in order to provide safe, affordable and quality surgical care. Specifically, the Lancet Commission recommended strategic health planning through the development of national surgical, obstetric and anaesthesia plans (NSOAPs) to be incorporated into national health policy, strategy or plans.

NSOAPs create a roadmap for improving surgical care through a country‐driven process that identifies gaps in current care, proposes solutions to bridge those gaps, and matches these with time‐bound targets to evaluate progress. Surgical care is complex; it needs the right workforce, with the right skills, equipment and infrastructure to come together at the same time^6^. Given this complexity, the plan can simultaneously address the six major building blocks of a health system: infrastructure (medical products, technology), workforce, service delivery, information management, finance and leadership/governance^7^. This transformational approach seeks to change the nature of surgical care development away from isolated vertical programmes towards a health systems approach, which is driven by national governments.

Given these advantages, an increasing number of countries have undertaken creation of national policies designed to strengthen the surgical health system^8^, ^9^, ^10^. There have been some early successes with NSOAP development resulting in partnerships between ministries of health, WHO, non‐governmental organizations (NGOs), industry, and academic institutions around the globe collaborating in the co‐creation of NSOAPs and mobilization of domestic resources to fund scale‐up of surgical care^9^, ^11^. This paper describes a possible skeleton process for developing a NSOAP from the authors' experiences developing some of the world's first NSOAPs in low‐ and middle‐income countries, discusses specific challenges encountered, and highlights lessons learned from navigating this process. It includes suggestions from Ministry of Health (MoH) officials, surgeons, practitioners, educators and consultants with experience in health system strengthening, health system planning and, specifically, NSOAP development. It aims to serve as a starting point and guide for countries looking to embark on this process while understanding that the process is fluid and adaptable to the given context.

## NSOAP development process

### General principles

**Figure 1:**
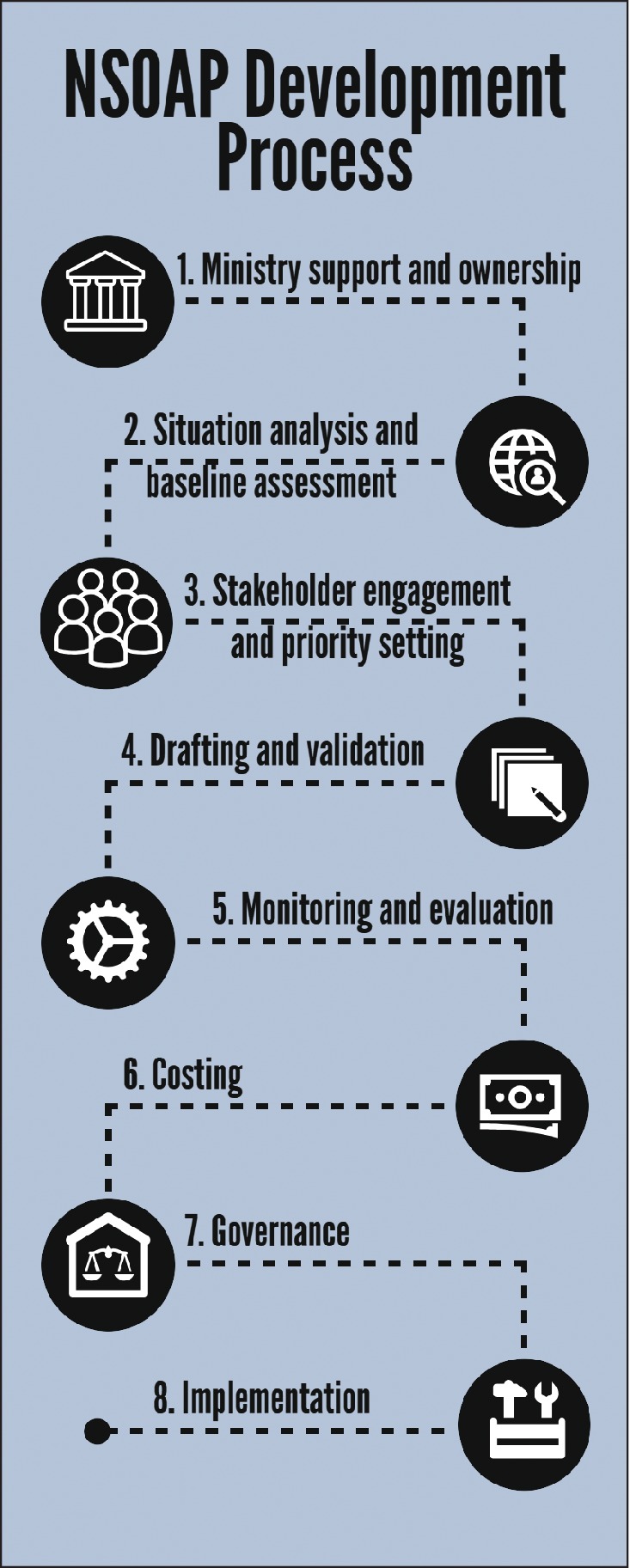
Steps for developing a national surgical, obstetric and anaesthesia plan NSOAP, national surgical, obstetric and anaesthesia plan.

The eight recommended key steps for developing a NSOAP are shown in *Fig*. 1. These steps were first developed during the NSOAP process in the Republic of Zambia (completed May 2017) and have since been adapted and agreed upon by a group of experts during the first NSOAP workshop held in Dubai, United Arab Emirates, in March 2018, with representation from 23 countries^8^, ^11^. Other countries, including Tanzania and Rwanda, have adopted a similar process, expanded upon to fit their own needs in developing their NSOAPs. *Appendix* *S1* (supporting information) describes the NSOAP process undertaken in Ethiopia.

### Ministry support and ownership

MoH support and ownership is arguably the most important for NSOAP development^12^, ^13^. Engaged MoH leaders are able to generate a well informed, realistic NSOAP and ensure smooth incorporation into the National Health Policy, Strategy and Plan (NHPSP). The MoH will provide an understanding of the current health priorities of the country, known gaps in broader healthcare, ongoing initiatives to address these gaps, financial capacities and implementation capabilities. For other stakeholders, MoH involvement provides a direct avenue to voice concerns to those most in power to influence policy and health sector priorities around SOA care. Finally, MoH ownership allows the creation of governance and accountability over the NSOAP, which can be integral to implementation^14^.

In many cases, the concept of NSOAP will need to be presented and advocated for to the MoH by experts in surgery, obstetrics, anaesthesia and public health who best understand the need for scale‐up of SOA care. Strategic planning often requires these ‘champions’ to advocate and push for the completion of the NSOAP^15^, ^16^. The champion provides leadership and motivation throughout the process, and is one of the driving forces for completion. Once the MoH has committed to NSOAP development, the creation of a lead team, including a broad group of individuals (such as society members, champion, influential providers, MoH and foreign partners), has previously been successful (Tanzania, Rwanda and Zambia) to drive the process, coordinate meetings, set agendas, and follow up on tasks^17^. Initially, the lead team can begin by drawing up a plan for the course of the NSOAP, as well as agreed roles and responsibilities for each team member^17^, ^18^. Time‐bound steps and determination of an overall timeline are encouraged to avoid loss of interest and momentum. The timing of NSOAP completion in relation to the budget cycle has been found to be critical to ensure that the first years of NSOAP implementation can be included within the budget cycle of the correct year; otherwise the NSOAP can lose momentum at a critical time after launch, while waiting for the new financial cycle.

It is then advisable for the lead team to define the scope of the NSOAP. This may be iterated during priority‐setting; however, an initial decision on the level of detail and the breadth of the plan is helpful to frame the steps required. Strategic plans are designed to provide a framework for scale‐up rather than to deliver step‐by‐step operational plans^13^. To execute each activity within the plan, more detailed programme planning will be needed with adjusted budgets based on real allocations and disbursements at the time of implementation.

Finally, in the early phases, it is also worth considering involvement of an expert consultant to provide additional administrative, technical and leadership support. The WHO Regional Office can provide technical expertise in strategic planning, costing, monitoring and evaluation, and international advocacy. Further, WHO can support the convening of major stakeholders, provide connections to other multilateral organizations, and assist in priority‐setting at the national level that synchronizes with WHO priorities^19^, ^20^.

### Situational analysis and baseline assessment

**Figure 2:**
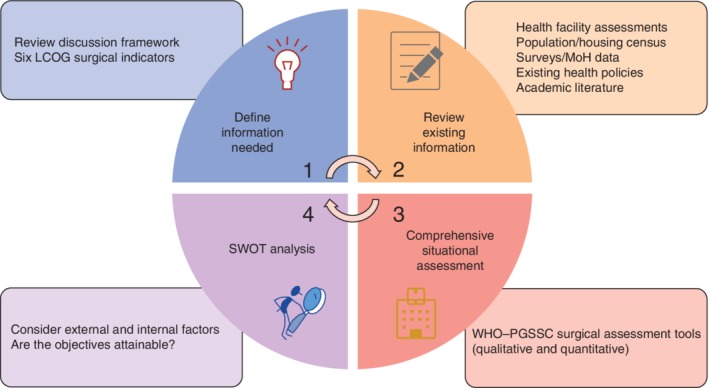
Situational analysis and baseline assessment phases LCOG, Lancet Commission on Global Surgery; MoH, Ministry of Health; SWOT, strengths, weaknesses, opportunities and threats; PGSSC, Program in Global Surgery and Social Change.

The next proposed step in the NSOAP process is to assess and understand the current surgical, obstetric and anaesthesia (SOA) landscape. A situational analysis and baseline assessment establish a consensus on the current gaps in care, allow for evidence‐based priority‐setting, and provide a baseline against which to compare future results. The baseline feeds into an overall monitoring and evaluation strategy, which facilitates accountability and transparency. Conducting a situational analysis can be thought of as involving four phases: define what information is needed; review existing information; gather additional information needed; and conduct a strengths, weaknesses, opportunities and threats (SWOT) analysis (*Fig*. 2).

The first phase encompasses identifying the information needed to best inform the priorities of the plan. The Program in Global Surgery and Social Change at Harvard Medical School created a discussion framework to help guide the discussions on priority‐setting^21^. Review of these will hopefully help to ascertain the information required for each domain (infrastructure, service delivery, workforce, information management, finance and governance). One consensus recommendation from thought leaders on NSOAP development was that baselining activities include assessment of the six surgical indicators that measure the strength and preparedness for delivery of surgical services, volume and outcomes of service, and financial risks to surgical patients (*Table* 1)^22^, ^23^, ^24^. Most of these indicators have since been accepted by the World Bank^25^ for inclusion into World Development Indicators, as well as by WHO^26^ in their 100 Core Health Indicators.

**Table 1:** Six surgical indicators to evaluate preparedness, service delivery and financial risk of a surgical system

Indicator no.	Indicator	Domain	Definition	Target 2030
1	Access to timely essential surgery	Preparedness	Proportion of population that can access, within 2 h, a facility that can do caesarean delivery, laparotomy and treatment of open fracture (Bellwether procedures)	80% coverage of essential surgical and anaesthesia services per country
2	Specialist surgical workforce density		No. of specialist surgical, anaesthetic and obstetric physicians working per 100 000 population	100% of countries with at least 20 surgical, anaesthetic and obstetric physicians per 100 000 population
3	Surgical volume	Service delivery	Procedures done in an operating theatre, per 100 000 population per year	100% of countries tracking surgical volume; 5000 procedures per 100 000 population
4	Perioperative mortality rate		All‐cause death rate before discharge in patients who have undergone a procedure in an operating theatre, divided by the total number of procedures, presented as a percentage	100% of countries tracking perioperative mortality
5	Protection against impoverishing expenditure	Financial risk	Proportion of households protected against impoverishment from direct out‐of‐pocket payments for surgical and anaesthesia care	100% protection against impoverishment from out‐of‐ pocket payments for surgical and anaesthesia care
6	Protection against catastrophic expenditure		Proportion of households protected against catastrophic expenditure from direct out‐of‐pocket payments for surgical and anaesthesia care	100% protection against catastrophic expenditure from out‐of‐pocket payments for surgical and anaesthesia care

The second phase, a comprehensive review of existing information, includes exploring MoH data (health sector management and information system, for example), national population and census data, nationwide surveys and facility assessments including, but not limited to, the Service Availability and Readiness Assessment, Demographic and Health Survey, Service Provision Assessments, Personnel, Infrastructure, Procedure, Equipment and Supplies, and Living Standards Measurement Study. A comprehensive search of the published academic literature can also provide a great deal of breadth and depth to the available information. Attention to past strategies within the country around SOA care can identify the successes and failures of past programmes and help guide future programme development. Review of existing MoH policies that may overlap with the NSOAP can help narrow the scope of the NSOAP and ensure that any decisions build upon, rather than duplicate or conflict with, existing strategies. Leveraging these existing sources of information reduces duplication of efforts and can focus the on‐the‐ground assessment on the questions yet to be answered. If additional or more up‐to‐date understanding is still required after the review of existing data and literature, a comprehensive situational assessment is encouraged.

In partnership with WHO, the Program in Global Surgery and Social Change has developed both a qualitative and a quantitative surgical assessment tool to aid in this phase^21^. The tool was developed through Delphi consensus with a multidisciplinary team that included surgeons, anaesthetists and obstetricians. The qualitative tool can be used for semistructured interviews with hospital directors, providers, nurses and other stakeholders to describe the current situation and opportunities at the facility level and in the SOA system more broadly. The quantitative tool analyses a hospital's current infrastructure, service delivery, workforce, information management and finance capabilities. For most countries, an on‐the‐ground comprehensive review of all facilities is difficult, given time and financial constraints. As an alternative, sample assessments of facilities across different hospital levels and regions have been undertaken. Although on‐the‐ground assessments are the standard in terms of validity, they can be supplemented by e‐mail or telephone surveys to overcome resource constraints^27^. Finally, a SWOT analysis is useful before developing a NSOAP to assess and consider both internal and external factors that may influence implementation of the NSOAP^14^, ^28^, ^29^. *Appendix* *S2* (supporting information) outlines the situational analysis used to tailor the NSOAP in Rwanda.

### Stakeholder engagement and priority‐setting

**Figure 3:**
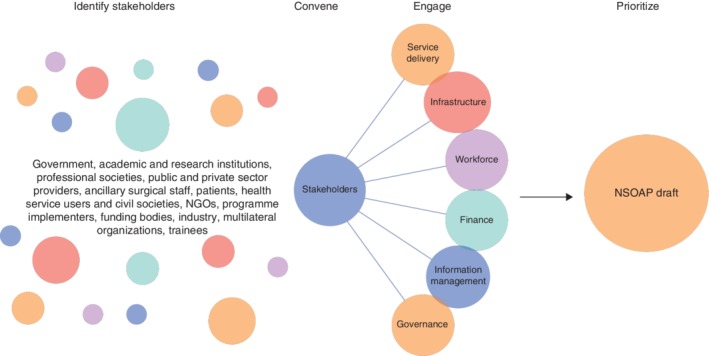
Stakeholder engagement and prioritization NGO, non‐governmental organization; NSOAP, national surgical, obstetric and anaesthesia plan.

Broad stakeholder involvement has been shown in the literature^30^, ^31^, ^32^, ^33^ to create comprehensive, transparent, feasible and trusted health policy. Further, there is a global recognition that best practice recommends a bottom‐up approach when discussing health system development and implementation^30^, ^34^, ^35^. Explicitly, a bottom‐up approach focuses on engaging stakeholders who are on the frontline of health services (such as physicians, ancillary staff, information technology specialists and engineers) who will ultimately be responsible for implementation of the NSOAP. As such, it is recommended to identify, engage and consult with stakeholders including, but not limited to, the following groups: government, academic and research institutions, trainees, professional societies, public and private sector providers, ancillary surgical staff, patients, health service users and civil societies, NGOs, programme implementers, funding bodies, industry and representatives of multilateral organizations (*Fig*. 3). The lead team can then discuss which groups to include, and can consider performing a stakeholder analysis to understand further what each group will add to the NSOAP and their level of involvement based on their expertise, power, influence and interest^36^. This initial broad stakeholder engagement serves to: ascertain the interests, priorities and concerns of each stakeholder group to ensure they are represented; raise awareness that a NSOAP process is underway; and identify individuals to serve as champions and become more deeply involved.

Following the initial identification and early engagement, a formal convening of core stakeholders can contribute to initial priority‐setting. Engaging with stakeholders can take many forms, including focus groups, semistructured interviews and workshops. It has previously been found useful to divide all stakeholders into separate committees, one for each of the six domains, with each committee having cross‐sectional representation of each stakeholder group. For example, having clinical providers represented in each of the six domains is beneficial for clear reasons, Ministry of Finance individuals are useful not only in the finance domain, but also in terms of infrastructure and governance, and so on.

The goal for each domain committee is to review the relevant situational analysis report, identify gaps in care and propose solutions. These solutions can then be prioritized based on their likely impact and effectiveness. Metrics and monitoring systems may then be developed for accountability (*Table* *S1*, supporting information). As mentioned above, a discussion framework (pgssc.org/national‐surgical‐planning) has been created for each of the domains to help guide these discussions and ensure the breadth of topics is covered^21^. Proceedings from these meetings can be compiled into a final report. To ensure cohesion across the domains, and that priorities in each domain are not being developed in isolation of one another, interim meetings between the differing domain committees are recommended. Once the reports have been compiled, a further round of consensus‐building and prioritization, taking into consideration the cost‐effectiveness, equity and health impact of each proposed activity, can further solidify appropriate priority‐setting. By identifying major themes across all domains, supported by evidence as well as experience, decision‐making on priorities can be rigorous and transparent. *Appendix* *S3* (supporting information) describes stakeholder engagement during Tanzania's NSOAP process.

### Drafting and validation

Once a final list of objectives and priorities has been agreed upon, the plan is ready to be drafted. The plan ideally follows the format of existing ministerial plans. To ensure this, it is helpful to include policy‐writers. Clearly defining the gaps and challenges identified from the situational analysis and baseline assessments gives a strong statement of the vision and mission for the NSOAP. The detail of the plan can then provide strategic objectives, goals and specific activities, and time‐bound targets, prioritized over a given period. For the first drafting of the plan, it is not practical to include all stakeholders; instead a smaller writing team with cross‐sectional representation of all stakeholder groups can be formed. This writing team is then tasked with combining all major conclusions from the previous steps and formulating a plan that reflects the views of all stakeholders, is evidence‐based, and aligns with the priorities of the government/ministry. Another practical solution is to task one or two people with developing a draft, which is then thoroughly reviewed line by line by the core writing team.

Developing detailed goals, strategic objectives, outcomes and activities is the most important part of a NSOAP, and deserves further explanation. Each domain requires goals, or broad statements of aim, for example ‘to increase SOA providers nationally’^37^. Under each goal, strategic objectives can then be developed to achieve that goal (for example ‘increase the number of surgeons trained at the university hospital’). From each strategic objective will stem the expected outputs (or outcomes) required to attain these objectives. Finally, each output can then have specific activities (or actionable items) to achieve an output, for example accrediting hospitals to take additional residents or recruitment into SOA specialties. Time‐bound targets can then be created.

Once the initial draft is complete, it can be iterated by the writing group before circulating to the wider group of stakeholders and community for feedback and validation. This ensures the NSOAP is an accurate and attainable representation of the goals, objectives, outputs, activities and targets discussed by stakeholders.

### Monitoring and evaluation

Having agreed upon priorities and activities, a thorough monitoring and evaluation plan is essential for successful implementation. Collecting data on surgical capacity and quality is the first step to improvement. Data can provide feedback to facilities and leadership involved in the SOA system from the bottom up, and create a metric for top‐down quality improvements. The data can be used for cost‐effectiveness analysis of policies to inform evidence‐based health policy decision‐making. As more countries develop NSOAPs, these indicators can be used for international comparison and benchmarking.

The selection of new indicators takes into consideration well established criteria of a good metric. They need to have a clear definition, be measurable, require reasonable resources to collect and analyse, and be within the control of the target users^38^. As well as the six Lancet Commission on Global Surgery indicators, a mix of fast‐moving targets within facility control (for instance on‐the‐day cancellation) on a quick feedback loop for motivation should be combined with slower‐moving structural targets (such as SOA density). Quantitative, measurable, time‐bound indicators for each activity, once defined, can then be integrated into existing data collection mechanisms within the overall national health information system, and collected at the health facility level. Creation of specific tools and a data flow plan for each indicator can also be discussed to ensure a complete feedback loop of data from facility up to the MoH, and from the MoH back to the facility. However, the time and detail required to define these additional tools and data flows may mean that this is best left to a dedicated team as an activity within the NSOAP. Where possible, a set target for each indicator should be listed. These targets can be absolute (numbers), relative (when the change from the baseline is unclear), or described as the annual rate of change (for example, a 3 per cent increase in the number of surgeons in 7 years)^39^. Examples of indicators from the Republic of Zambia are shown in *Table* 2.

**Table 2:** Service delivery indicators, base and targets from the Republic of Zambia's national surgical, obstetric and anaesthesia plan

				Target	
Results chain	Indicator	Means of verification	Base	Mid term 2019	End term 2021
Output 6: Establish and strengthen the provision of quality comprehensive, highly specialized, and complex surgical case at level 2 and 3 hospitals	No. of facilities providing pain management services on labour wards		0	18	36
	No. of facilities providing paediatric anaesthesia		6	18	36
Output 6: Establish and strengthen the provision of quality comprehensive, highly specialized, and complex surgical case at level 2 and 3 hospitals	No. of facilities providing neonatal anaesthesia	Level 2 and 3 hospital annual reports, MoH annual reports	2	3	7
	No. of facilities providing cardiac anaesthesia		0	1	2
	No. of facilities providing renal anaesthesia		2	3	4
	No. of facilities with functioning ICUs		4	8	16
	No. of facilities with functioning neonatal ICUs		1	2	2
	No. of facilities with HDUs		9	18	36
	No. of facilities that received and are utilizing admission and treatment protocols to ICUs/HDUs		0	8	16
	No. of facilities providing out‐of‐theatre anaesthesia		1	2	2

MoH, Ministry of Health; HDU, high‐dependency unit.

### Costing

The steps to costing are to assemble available costing information, defining the cost objects (activities, programmes, services, items needed, and so on) and quantities required, determining the cost base (local cost for each cost object), and attributing this cost to the appropriate number of cost objects.

Assembly of available cost information and determination of cost bases involves expertise from several sources. The Ministry of Finance can provide guidance regarding a realistic total budget given the current financial landscape. Other groups that may have considerable costing information are human resources, training, procurement and pharmacy departments within the MoH. Multilateral organizations, NGOs and other funding bodies with experience of funding the implementation of similar programmes may also have relevant information. It may be efficient to bring these parties together as a workshop to go through the steps defined above.

At the point of costing, a further round of prioritization of activities can occur, reviewing the cost‐effectiveness of interventions and how realistic each activity seems given the total overall budget. The specifics of how much of the projected costs is eventually allocated and disbursed will occur through annual parliamentary budget discussions and debate, and expectations of stakeholders should be managed to be clear it is unlikely that the entire plan will be funded outright by domestic funds from the first year.

Once costing is completed, like the finalization of the draft, the costing document may be presented to stakeholders, experts and community members for validation. There are multiple tools available to assist with the costing and prioritization process^21^, ^40^. A strategically and thoughtfully costed plan can be used in discussions with external funding bodies and implementation partners to improve advocacy^41^.

### Governance and implementation

Governance broadly refers to the rules, laws and organizational structure required to assist an organization in achieving the objectives of its strategic plan^42^. Health governance specifically includes mechanisms to promote health on the national agenda and establishment of transparent accountability mechanisms. In most countries, governance for the health sector is already well defined, and so the key is aligning NSOAP governance to the existing framework and mechanisms. Strong governance of the NSOAP will lead to improved visibility for SOA care, allowing for promotion on the national health agenda, better coordination of SOA care to other health sectors, and establishing cyclical communication and accountability mechanisms from the community and facility up to the national level and back. Given that NSOAP is designed for health systems strengthening, each activity and domain is co‐dependent. As such, coordination of implementation activities is essential for maximal impact. Implementation of scattered activities is unlikely to have a significant effect.

At the national level, it is recommended to establish early on whether the NSOAP will be within an already recognized department (reproductive, maternal and child health, or non‐communicable diseases, health services, quality departments, and so on) or merits a new department of its own. Given the breadth of NSOAP activities, it is important to have dedicated NSOAP implementation personnel, ideally a NSOAP director, with an additional NSOAP coordinator for each of the domains at the country level. These individuals would be responsible for further strategy development, coordination of activities, leadership, reporting of progress, and resource allocation and mobilization. Below this lead team there can be a working group to assist and advise in prioritization of NSOAP implementation; this should include representation from each of the major stakeholder groups. At the regional level, depending on the country, it may be useful also to have a regional NSOAP director/coordinator who oversees the work of that region and acts as a bridge between the facility and Ministry.

Governance at the facility level is the cornerstone of implementation and success of a NSOAP. Each facility should ideally have a multidisciplinary NSOAP team (surgeons, anaesthetists, obstetric providers, nurses, midwives, and so on) led by a NSOAP champion for that facility. This team is responsible for devising facility‐level implementation projects for the NSOAP, mobilizing the necessary resources to achieve NSOAP goals, and ensuring accountability and execution of projects. It is suggested that the team hold scheduled monthly meetings to discuss progress and review collected NSOAP monitoring and evaluation data.

Before implementation, the plan should be widely disseminated within both the public and private health sectors, across medical academic institutions and within the community, so that the strategic framework is understood and unified in execution and thought. Plans should be shared at the international level to help guide other countries and spur conversation on common barriers and possible solutions (*Table* 3).

**Table 3:** Development pearls for national surgical, obstetric and anaesthesia planning

Ministry support and ownership is required, and is the key to success
Create a timeline for the process and stick to it
Appropriate baselining leads to appropriate targets
Broad stakeholder involvement from the frontlines will create a well informed plan
Address the hard issues, despite their complexity and burdensome nature
Decide on set indicators and incorporate into health management information system
Involve implementers and financing bodies throughout the entire process to ensure implementation

## Limitations

There are many ways to go about developing a strategic health plan, and these recommendations should therefore be taken as suggestions, based on challenges encountered and solutions identified while working on the development of a NSOAP in multiple countries^43^. These recommendations do not guarantee successful completion of a NSOAP document, but rather serve as a helpful guide. Further, implementation of NSOAPs is just beginning in the countries mentioned (Ethiopia, Zambia and Tanzania, and soon in Rwanda); therefore, the impact of NSOAPs on surgical outcomes, health system strengthening and sustainable scale‐up is still unknown. The real challenge remains in how the NSOAP document can or will translate into improved access to surgical care.

## Conclusion

The NSOAP approach has several key advantages. First, this nationally driven effort promotes visibility and accountability around SOA care. The NSOAP process itself requires mobilization of the national SOA stakeholders to come together towards a common vision of future surgical care delivery in their country. This grass roots mobilization, with a tangible early goal, helps create a movement to sustain collaboration between SOA stakeholders and the MoH for further advocacy and implementation. The creation of the plan itself highlights SOA care and demonstrates high‐level political commitment. Integration of the NSOAP into their NHPSP then ensures SOA care, previously widely neglected, as an integral part of the healthcare system^44^. Second, the plan allows SOA stakeholders to participate in national priority‐setting. This ensures the alignment of front‐line staff and government priorities. Defining these national priorities is the key to improved coordination of resources and resource allocation. With a clear national framework, domestic and international initiatives can build synergistically towards a common goal, rather than the current fragmented system with external priority‐setting. Finally, a clear roadmap with time‐bound targets helps to reinforce accountability to attract further domestic and international funds.

There is global movement to address the burden of surgical disease worldwide and to improve quality and access to SOA care. The development of a strategic plan to address systematically the gaps across the SOA system is a critical first step to ensuring countrywide scale‐up of activities that strengthen surgical systems.

## Disclosure

The authors declare no conflict of interest.

## Supporting information


**Table S1** NSOAP template of major domains, implementation strategies and indicators
**Appendix S1** Ethiopia's national surgical, obstetric and anaesthesia planning process
**Appendix S2** Comprehensive situation assessment in Rwanda
**Appendix S3** Stakeholder engagement in Tanzania
