# Supplementary material for: Framework for developing a national surgical, obstetric and anaesthesia plan
Source: BJS Open. 2019 Jul 24;3(5):722–32. doi: 10.1002/bjs5.50190 (PMC6773655; doi:10.1002/bjs5.50190)
Supplement: Supplementary file 1 — Table S1 NSOAP template of major domains, implementation strategies and indicators Appendix S1 Ethiopia's national surgical, obstetric and anaesthesia planning process Appendix S2 Comprehensive situation assessment in Rwanda Appendix S3 Stakeholder engagement in Tanzania [file BJS5-3-722-s001.docx]

**BJS5_50190**

**Framework for developing a national surgical, obstetric and anaesthesia plan**

**K. A. Sonderman, I. Citron, S. Mukhopadhyay, K. Albutt, K. Taylor, D. Jumbam, K. R. Iverson, M. Nthele, A. Bekele, E. Rwamasirabo, S. Maongezi, M. L. Steer, R. Riviello, W. Johnson and J. G. Meara**

**Table S1** NSOAP template of major domains, implementation strategies and indicators

| **Service Delivery** | | |
| --- | --- | --- |
| Components | Recommendations** | Indicators (or assessment methods?) |
| - Distribution and volume of services - Quality and safety - Perioperative services - Referral system and coordination - Community education and outreach - Service consistency | - Standard procedure lists for each facility level (all hospital to provide Bellwether procedures) - Quality control initiatives, eg. use of WHO checklist, peri-op antibiotic - Peri-operative standard operating procedures and standards - Referral protocols and data loops for transfer from community to tertiary level and back - Community education and outreach - Measures for service consistency and data loops - Ambulance and Patient transport services | - Proportion of facilities performing Bellwether - Proportion of population within 2hrs of Bellweather facility - 2-hour access* - Procedures per population* - Perioperative mortality rate* - Appropriateness of referrals/year/facility |
| **Infrastructure** | | |
| - Surgical facilities - Equipment, medicines and supplies - Equipment preventative maintenance and repair - Blood supply - Supply chain - Diagnostic and ancillary services - Sterilization, Laundry and waste management - Patient transport | - Ensure adequate number and distribution of facilities performing SOA care - Define equipment/medicine/supply list for each facility level and procure - Establish maintenance and repair of equipment and train necessary staff - Establish blood protocol and standards - Strengthen medical supply chain logistics and management - Set minimum standards for radiology and laboratory capabilities - Improve sterilization, laundry services and establish waste protocols | - Proportion of facilities adequately stocked/year - Blood bank distribution and supply - Adherence to waste protocols |
| **Workforce** | | |
| - SOA specialist density - Ancillary staff - Workforce Distribution and retention - Education and training/credentialing - Employment - Continued medical education - Healthcare management | - Based on population needs, set training strategy and targets for SOA specialist and subspecialist providers and specialist ancillary staff (e.g BMET, physiotherapy, nursing, radiology, pathology …) - Develop strategies to ensure improved distribution and retention of providers including rural components of training, bonding, cluster distribution of staff to avoid burnout, improved CME, improved financial and other incentives. - Establish training and credentialing of non-specialist providers in basic surgical procedures - Supervision programs for general physicians, anesthesia techs, etc. - Create CME programs, required credits, and governing bodies - Expand and professionalize healthcare management | - SAO density per population* - SAO density in rural areas - Change in number of providers/year - Non-specialist credentialing - Adherence to CME required credits |
| - **Information Management** | | |
| - Health system indicators and data collection - Registries - Research, outcomes monitoring - Data leveraging and usage | - Strategic plan for data collection around process’, outcomes and expenditure at both the private and public level - Creation of trauma, cancer registries - Identify, regulate and fund surgical research priorities of local relevance - Establish designated research centers mentors and opportunities - Develop information management systems to track data collection and reporting | - % of facilities prospectively registering surgical data - Data quality of registries |
| - **Finance** | | |
| - National and hospital budgets for surgical services - Patient cost of surgical care - Universal health coverage (UHC) - Advocacy | - Track budgets the facility and national level for SOA care - Track and monitor out of pocket payments (OOP) for surgical procedures - Establish surgical package of procedures covered under UHC - Advocate for additional domestic and development assistance funding for SOA care | - Surgical expenditure as a proportion of GDP - Surgical expenditure as a proportion of national health care spending - Catastrophic and impoverishing expenditure* - Out-of-pocket expenditures on surgery |
| - **Governance** | | |
| - Leadership and accountability - Coordination of activities - Terms of engagement - Monitoring and evaluation | - Hire national NSOAP coordinator, lead team, and designate NSOAP personnel at each level - Each facility to have a multidisciplinary theatre user group to review Monitoring and Evaluation data - Ensure detailed action plan for each activity, coordinating with other health sectors - Establish roles of Ministry departments, Professional societies and other non-state actors - Each activity to have a M&E plan with review process | - Tracking of NSOAP coordinators/facility participation - Number of facility reports reviewed/year |

[*LCOGs surgical indicators](https://www.pgssc.org/national-surgical-planning); [**Comprehensive list of implementation strategies](https://www.pgssc.org/national-surgical-planning)

**Appendix S1** Ethiopia’s national surgical, obstetric and anaesthesia planning process

The NSOAP development process in Ethiopia began in 2014. Ethiopia has an unmet need of five million surgical operations per year, surgery waiting times of up to 4 years, and a severe shortage of SOA providers. The Saving Lives Through Safe Surgery (SaLTS) initiative was designed to improve equitable access to quality and safe essential and emergency SOA care as part of universal health coverage. Eight intervention pillars were selected: leadership and governance; human resources; partnerships; drugs, medical equipment and supplies; infrastructure development; quality; innovation; and monitoring and evaluation. Specific targets and activities were established for each of the pillars. Key factors underpin the early success of the SaLTS initiative in Ethiopia. The government has ownership of the initiative, provides strong leadership, and has committed significant domestic resources. The project has a clear governance framework for dissemination from the Ministry of Health to facilities and back. The initiative also recognizes the importance of partnerships and collaboration at the local, national and international levels. The lessons gleaned during experiences, particularly in governance and monitoring and evaluation, are being applied to national scale-up. Challenges include the lack of uniformity in implementation throughout all regions in the country, attracting new partners and investors to continue implementation, ensuring robust evaluation of early results, and maintaining the early-stage momentum of the initiative.

**Appendix S2** Comprehensive situation assessment in Rwanda

Rwanda performed a thorough situational analysis to facilitate the development of a strategic, data-driven plan. First, a thorough review of the published literature on surgical capacity and national data on SOA care was summarized. Next, over a 3-week period, all 42 public district hospitals were surveyed utilizing the WHO–PGSSC surgical assessment toils (both qualitative and quantitative). The surveys were conducted by current surgical residents from the University of Rwanda. Hospital data and log books were reviewed to calculate surgical volume per population, ability to perform Bellwether procedures (caesarean section, laparotomy, open fracture repair), tracking of perioperative mortality, and surge SOA density for district hospitals. Further detailed data were collected around infrastructure, service delivery, workforce, finance and information management at each hospital. The data were then analysed for review by the Ministry of Health and other major stakeholders. The situational analysis was used to tailor the NSOAP, to address the specific gaps identified.

**Appendix S3** Stakeholder engagement in Tanzania

Tanzania’s NSOAP process began in November 2016 and finished in March 2018. First, a situational analysis was performed by carrying out a systematic review of all existing data from the Ministry of Health, non-governmental organizations (NGOs), grey literature, policies and academic publications. Next, more than 200 diverse stakeholders were consulted, identified through a snowball approach, and engaged through semistructured interviews and focus groups. The major stakeholder groups included:

– Clinicians (surgeons, anaesthesiologists, anaesthetists, obstetricians, radiologists, nurses, task-sharers, laboratory technicians, biomedical engineers and midwives) from each zone of Tanzania

– Civil societies, NGOs and patient groups

– Government (curative and preventive services, policy and planning, human resources, training, procurement, etc.)

From this large group of stakeholders, a total of 80 participants were selected for a 2-day technical workshop. Using the discussion framework this group identified priorities, which were synthesized into a draft and validated by different groups. The plan was then costed (estimated at US$1.7 per capita per year, representing 3.28 per cent of current health spending); it included 150 individual activities.
